# Biomechanical effects of Teuscher activator in hyperdivergent Class II malocclusion treatment: A finite element analysis

**DOI:** 10.4317/jced.58722

**Published:** 2021-11-01

**Authors:** Marta Jorge, Mário Vaz, Jorge Lopes, Josep-Maria Ustrell-Torrent, Behzad Farahani, Maria-João Ponces

**Affiliations:** 1 PhD Student of Faculty of Dental Medicine of University of Porto; 2 Associate Professor with Tenure, Faculty of Engineering; 3 Full Professor of Faculty of Dental Medicine of University of Porto; 4 Full Professor of Vice Dean of Dentistry of Faculty of Medicine and Health Sciences. University of Barcelona. Oral Health and Masticatory System Group (Bellvitge Biomedical Research Institute) IDIBELL; 5 Post Doctoral Researcher in INEGI - Instituto de Ciência e Inovação em Engenharia Mecânica e Engenharia Industrial; 6 Auxiliary Professor of Faculty of Dental Medicine of University of Porto

## Abstract

**Background:**

In orthodontic treatment, the combination of an activator with a headgear is commonly used in treatment of the hyperdivergent Class II malocclusion. However, the distribution of stresses transmitted to the maxilla by these appliances has been little studied. This study aimed to compare the biomechanical effects of stresses transmitted to the maxilla and teeth by a Teuscher activator (TA) for different lines of action of extraoral force, using finite element analysis.

**Material and Methods:**

A tridimensional finite element model of the maxilla and teeth was created based on the true geometry of a human skull. The (TA) and the face bow were designed in 3D computer-aided design and fixed in the maxilla model. To study the effects of mechanical stress transmitted to the maxilla in the treatment of hyperdivergent Class II malocclusion with (TA) combined with extraoral forces, five different finite element models were used, considering the centers of resistance of the maxilla and dentition.

**Results:**

The results showed that stresses increased progressively when the force line of action moved in posteroanterior direction. Von Mises equivalent stress was lower in Model 1 (0°) than in Model 5 (60°). In Models 1 (0°) and 2 (15°), molars suffered greater distal displacement and incisors showed extrusion. In Model 3 (30°), the force line of action promoted a distal displacement of molars and incisors. In Models 4 (45°) and 5 (60°), the whole maxillary anterior sector showed counterclockwise displacement.

**Conclusions:**

Different force lines of action influence the intensity and distribution of orthodontic and orthopedic forces in the maxilla. The extraoral force’s line of action used in Model 3 (30°) is the most compatible with the objectives of the hyperdivergent Class II malocclusion treatment in growing patients.

** Key words:**Class II, Headgear, Early treatment, FEA.

## Introduction

Class II malocclusion may be caused by dental or skeletal maxillary protrusion or both. Patients with Class II malocclusion and the hyperdivergent phenotype usually suffer a variable combination of skeletal and dentoalveolar changes in the three spatial planes. They also have retrognathic mandibles, long posterior and anterior dentoalveolar facial heights, increased gonial angles and mandibular planes, among other changes (1,2).

The most common and least invasive approach in the early treatment of this condition has been using high pull extraoral force when maxillary displacement restriction, distalization, and maxillary molar intrusion are important goals for sagittal and vertical correction and facial profile improvement (3-11).

Nevertheless, several studies have reported unwanted effects of using functional appliances combined with extraoral forces for vertical control, namely, the partial restriction of the maxilla’s anterior displacement, increased anteroinferior facial height, and posterior rotation of the mandible however, those studies are not consensual (8,12-14).

In hyperdivergent Class II malocclusion treatment, understanding the tridimensional (3D) effects of biomechanical stress transmitted to the maxilla, namely to teeth and mid-facial skeletal structures, is crucial to identify the best force line of action for better vertical control at the maxillary level.

Few studies focused on explaining the effects of the dissipation of biomechanical stress transmitted to the maxilla by functional appliances combined with extraoral forces used in hyperdivergent Class II malocclusion treatment (15,16).

The use of finite element analysis FEA has been a useful tool in the evaluation of biomechanical effects, such as displacements, strains and stresses induced in living structures by external forces and is considered an asset in predicting the effects of orthodontic treatment (17-20).

Therefore, this study aimed to use finite element analysis FEA to compare, in these patients, the biomechanical effects of stresses transmitted to the maxilla by a Teuscher activator (TA) with different directions of the extraoral force.

## Material and Methods

-Model Configurations: Maxilla and Teeth 

A 10-year-old female patient with Class II, division 1 malocclusion and a hyperdivergent Class II skeletal pattern was selected for this study. She had not been subjected to any previous orthodontic treatment. A dentulous human maxilla obtained from the Grab CAD database was used as a reference.

A 3D computer-aided design (CAD) model of the patient’s maxilla, including teeth, was created based on images of the patient obtained from DICOM (digital imaging and communication in medicine) data in computed tomography (CT) format. The use of these images to create the model was approved by the Ethics Committee of the Faculty of Dental Medicine of the Porto on May 5, 2018, under registration number 527. The model had to be adjusted to the patient’s dimensions for agreement between the numerical model and the clinical case. Measurements were made on the physical model using a dial-caliper (Absolute Digimatic, Mitutoyo).

The model was then processed using the CAD software SolidWorks® (Dassault Systems SolidWorks Corp., Concord, MA, EUA), and all CAD data were adapted to the patient’s anatomy. This study focused on the maxillary region, limited superiorly by the orbital floor and posteriorly by the pterygomaxillary suture. The final model was composed of the maxilla, the skull base (zygomatic, nasal, and sphenoid), the incisors, and the maxillary first molars. Due to the patient’s age, the permanent premolars were absent at treatment onset.

-Model ConFigurations: Teuscher Activator and Face Bow 

 A (TA) combined with a face bow was incorporated in the maxillae and teeth’ anatomical model to represent clinical conditions. A 3D model of the (TA) and face bow was developed using the SolidWorks® software, based on images of the physical model and measurements obtained with the dial-caliper.

The (TA) consists of an acrylic monobloc that surrounds the whole occlusal and palatal aspects of teeth up to the distal level of the maxillary first molar and about 2 mm of their buccal aspect. Superiorly, at the palate level, it has a Coffin spring made of steel wire (diameter: 0.09 mm). Its anterior maxillary portion has four springs made of steel wire (diameter: 0.08 mm) to offer torque to the maxillary incisors and some retention to the (TA) when inserted in the dental arch. Laterally, at the level of the primary second molar, two metallic tubes attached to the TA’s acrylic accommodate the face bow’s inner bow.

The face bow consists of a stainless-steel arch (diameter: 1.1 mm) with two bows: the inner bow and the outer bow. The inner bow enters a metallic tube laterally attached to the TA’s acrylic at the level of the primary second molar.

In our model, the outer bow assumed different angulations, taking into account the center of resistance of the maxilla (CResM) and dentition (CResD), and five different lines of action of extraoral force were applied.

The (TA) was modeled as a simple acrylic bloc, and each incisal edge and cusp of the teeth was well inserted into the acrylic. The outer bow’s geometry, where force is applied (hooks), was modeled. However, to simplify the numerical simulation, hooks were not considered.

**Figure 1:**
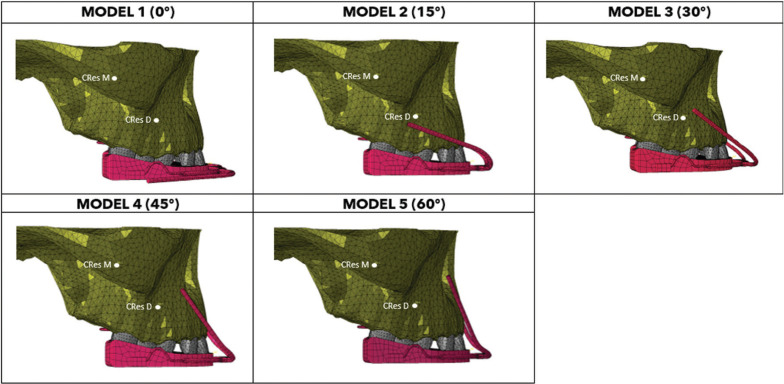
Finite element model where the force lines of action used for each of the five models are indicated (M1 (0°), M2 (15°), M3 (30°), M4 (45°) and M5 (60°).

Numerical studies were conducted using FEA simulated in Abaqus® in the static time step regime to assess stress distribution. The maxilla’s anatomical model and the (TA) with the face bow were imported to the FEA model and five (finite element) models were created to simulate the application of five different force lines of action (Fig. 1).

The finite element mesh included a total number of 37.326 and 150.781 nodes and elements, respectively. In which 145.313 linear tetrahedral elements of type C3D4, 4018 linear quadrilateral elements of type S4R and 1.290 linear triangular elements of type S3 built the mesh.

-Materials’ Boundary Conditions and Properties 

The mechanical properties of each part of the model were defined using young’s modulus and Poisson’s coefficient. Every material was assumed as homogeneous, isotropic, and linearly elastic. The boundaries of the bone’s cortical and cancellous layers, enamel, and dentin were not considered in this study to facilitate the creation of the finite element mesh and simplify the model. Thus, a single value was used to represent both properties. The mechanical properties used for the teeth, bone, and (TA) have been reported in the literature (21) and are summarized in Table 1.

**Table 1:**
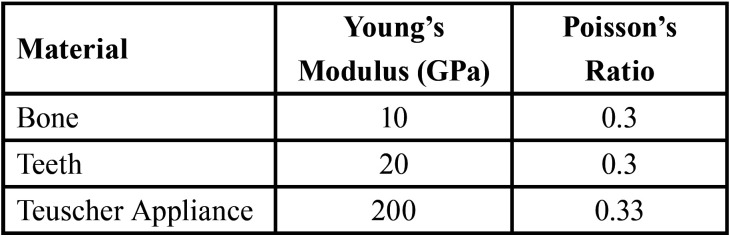
Mechanical properties of the materials: Young´s modulus and Poisson´s coefficient.

The (TA) and the face bow were considered a rigid body and modeled as a single unit. Rigid bodies are particularly effective for modeling relatively rigid parts of a model in Abaqus®, especially when the tissues’ mechanical properties are significantly inferior to those of the materials that compose them.

The force’s magnitude was selected based on clinical situations, according to the literature (16). A 4.4N (450 g) load was applied on each side of the geometric model, creating five models to simulate five different force lines of action (Model 1 (0°), Model 2 (15°), Model 3 (30°), Model 4 (45°), and Model 5 (60°) (Fig. 1).

The model’s boundary conditions were set according to the junction between the maxilla and the cranial bone structures. Accordingly, the geometric model was fixed on the maxillary (skull base) and posterior (pterygoid pillar) surfaces, hence preventing displacement or rotation in any direction. Tight contact was assumed in the interfaces between the different parts of the model. Stress distribution in the five models studied was estimated by linear static analysis.

**Figure 2:**
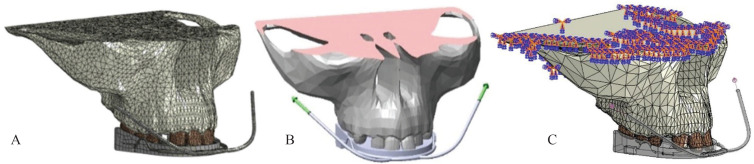
Finite element model: A. Mesh. B. Applied loads. C. Boundary conditions.

A mesh convergence study was conducted based on the von Mises stresses (23). Von Mises stress results, estimated at an approximate midpoint of the maxilla and in a uniform-stress region, converged as the mesh density increased. Considering the geometric complexity, the mesh convergence study allows evaluating the quality of the approximation obtained (Fig. 2).

## Results

The objective of creating a maxillary biomechanical model to simulate Class II malocclusion treatment using a (TA) combined with a face bow was achieved. Von Mises equivalent stress was selected as the parameter for outcome evaluation (23).

A color scale was used, where red colors indicate areas subjected to a high-stress peak, while blue colors reflect stress levels close to zero. The main focus of the results was the concentration of stress transmitted to the maxilla by the (TA) with five different force lines of action (Fig. 1).

**Figure 3:**
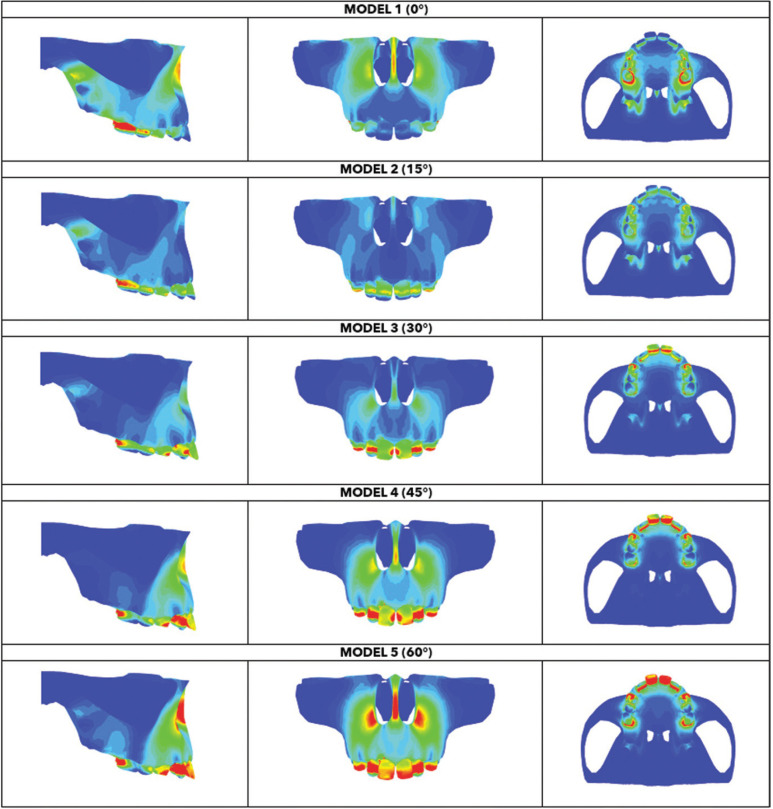
Von Mises stress distributions obtained from different models by FEM, values are in MPa.

The results showed that different force lines of action interfered with stress distribution in the bone structures (Table 2). In every model tested, the highest stress concentration was found in the frontal region. The maxilla’s anterior region, near the incisor foramen, showed slight stress dissipation through the palate in the anteroposterior direction. This stress distribution pattern was found in every model, but the maximum stress intensity varied (Fig. 3, Table 2).

**Table 2:**
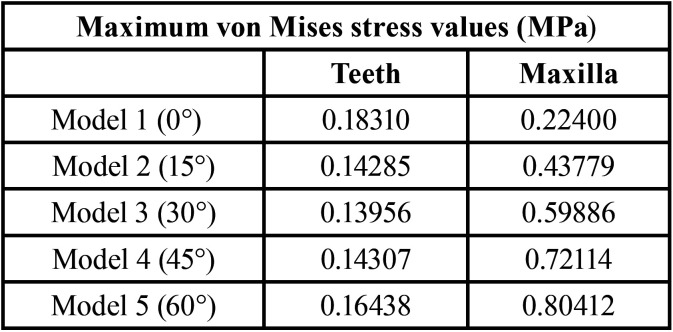
Maximum von Mises stress values.

Model 5 (60°) induced the highest stress concentration, as observed in (Fig. 3). Conversely, stress concentration was lowest in Model 1 (0°). Stress increased progressively when the force line of action moved in a posteroanterior direction. Model 1 (0°) showed that stress concentration was highest at the molars, the nasal bone (nasal septum and pyriform aperture limits), and the pterygoid fossa. Despite showing the lowest stress concentration, this model showed increased stress distribution in an anteroposterior direction. In Model 2 (15°), the highest stress concentration occurred at the molars, and stress at the incisors was higher than in Model 1 (0°). However, stress concentration at the nasal septum was lower. In Model 3, stress concentration was highest at the molars. Stress at the incisors was higher than in the previous models and was distributed in the frontal region. Models 4 (45°) and 5 (60°) had similar stress distribution areas, but Model 5 (60°) showed the greatest distribution area and the highest stress levels compared to the other models. Due to force application, stress distribution was more similar between Models 1 (0°) and 2 (15°) and between Models 4 (45°) and 5 (60°), (Fig. 3).

Regarding individual dentoalveolar tooth behavior, results show higher stress at the incisors and molars than at the support region.

**Figure 4:**
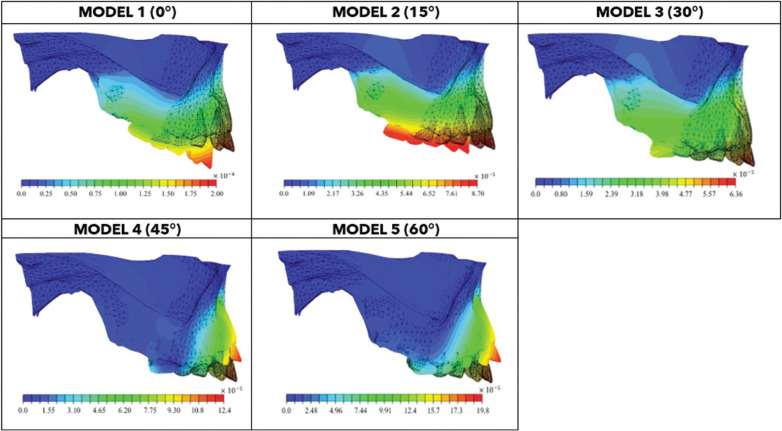
Displacement magnitude profiles captured on deformed and undeformed shape for different models extracted from FEM, values are in mm.

Although the applied force’s magnitude was similar in every model, in Models 1 (0°) and 2 (15°), molars suffered greater distal displacement and incisors showed extrusion. In Model 3 (30°), the force line of action promoted distal displacement of molars and incisors. In Models 4 (45°) and 5 (60°), the whole maxillary anterior sector showed counterclockwise displacement (Fig. 4).

## Discussion

FEA is based on a mathematical model whose geometry and boundary conditions are similar to the structure’s ones and considers the mechanical properties of each component of the model. It is basically a numerical calculation tool that divides continuous bodies into discrete elements – finite elements, with mechanical properties close to those of the tissues they represent. The results’ approximation depends inversely on the size and number of elements, which is why the convergence analysis was conducted von Mises. FEA may be useful to optimize oral structures and predict orthodontic treatments since in-vivo studies are difficult, time-consuming, and expensive. Moreover, numerical simulation has been reported as an effective tool for assessing the effects of different orthodontic appliances (17,18,20).

Force application in the maxilla creates differential stresses that may influence maxillary growth, thus being considered a valid approach for growing hyperdivergent patients with Class II malocclusion and associated maxillary protrusion (11,13,24). The effects of stress transmitted to the maxilla in this type of hyperdivergent Class II malocclusion treatment are extremely important but have been little studied. Thus, this study implemented FEA to assess the distribution of stress transmitted to the maxilla (teeth and maxillofacial complex) by a (TA) combined with five different lines of action of extraoral force.

Some parameters, including the force’s magnitude, point of application, and line of action, must be considered for obtaining excellent results when using extraoral forces (25,26-28).

Bowden *et al*., (25) in 1978, confirmed the importance of knowing the point of force application to better understand changes in palatal plane inclination. If the force vector passes through the maxilla’s center of resistance, no momentum is created, and the maxilla should not rotate. However, if the force vector does not coincide with the maxilla’s center of resistance, the maxilla is expected to rotate. In that case, the direction and the momentum created will depend on the shortest perpendicular distance between the force vector and the corresponding center of resistance.

In 1986, Teuscher showed that, when a high - pull force whose line of action coincides with the maxilla’s center of resistance (CResM) and the maxillary dentition’s center of resistance (CResD) is used, no rotations are expected, either by the maxilla or the maxillary dentition. However, if that force line of action passes between the CResM and the CResD, the maxilla rotates clockwise, and the maxillary dentition rotates counterclockwise. On the other hand, if the force line of action passes below the CResM and the CResD, both the maxilla and the maxillary dentition rotate clockwise (27).

In our study, a 4.4-N (450 g) force was applied on each side of the model with five different lines of action to identify the one that better suited the maxillary vertical and sagittal control in hyperdivergent Class II malocclusion treatment of a growing patient (26,27,29).

Using the extraoral force combined with the (TA) is particularly important because the forces are dissipated not only to the teeth but to every structure covered by the TA’s acrylic, contrary to what happens when the extraoral force is applied directly on the bands placed on maxillary molars.

In our study, despite all the maxillary dentitions being covered by the TA’s acrylic, the highest stress concentration was found at the level of the incisors and first molars. Some previous studies using FEA focused only on the application of high - pull extraoral forces directly on the maxillary first molars and showed some areas of stress on the root surface of the maxillary first molar (15,16,30).

A study conducted by Maruo *et al*., (16) modeled the maxilla, the maxillary teeth, and the headgear but did not consider the activator. They detected the highest displacement of the maxillary first molars with the low (cervical) pull, followed by the horizontal pull and the high pull. They also obtained greater intrusion of the maxillary first molar with the high pull, contrary to what was observed in our study (16). However, in our study, the (TA) was also modeled, besides the bone and every tooth, at treatment onset. Overall, the dynamics of the structures represented in that study do not intimately coincide with those presented in our models.

The materials’ properties considered in our study represent mean values that do not take into account the patient’s individual differentiation, age, gender, and diet. Moreover, the periodontal ligament was not considered to avoid any inconsistency and imprecision associated with modeling due to the differences between the periodontal ligament and the bone’s mechanical properties. Despite these limitations, the results obtained in this study are extremely useful due to providing pertinent information for orthodontists and allowing the optimization of clinical procedures. In fact, the impact the different force lines of action have on the clinical effect highlights the importance of this precise control to reach the results set in the treatment planning.

In our study, Model 3 (30°) was the most consistent with the clinical objectives of hyperdivergent Class II malocclusion treatment in a growing patient due to the posterior displacement of the teeth and the nasomaxillary complex. Thus, anterior and inferior displacements were limited due to normal growth, contributing to the correction of the skeletal discrepancy because of promoting maxillomandibular differential growth.

In Models 1 (0°), and 2 (15°), the force application resulted in a clockwise displacement of the whole maxillary complex, which is not desirable in hyperdivergent Class II malocclusion treatment since it results in undesirable increased anteroinferior facial height. In Models 4 (45°) and 5 (60°) the maxillary complex rotated counterclockwise with a posterior dental extrusion effect, which is not desirable in hyperdivergent Class II malocclusion treatment, as it promotes a posterior rotation of the mandible or, conversely, a need for condylar distraction inside the joint to allow for adaptative dental intercuspidation.

We hope that the present study promotes further studies that assess the effects of extraoral forces’ biomechanical stresses on both the maxilla and the mandible.

## Conclusions

In this study, a finite element model was built to simulate the TA’s effects on hyperdivergent Class II malocclusion treatment. The model was created based on a real anatomical geometry obtained by CT and the tissues’ mechanical properties reported in the literature. The tissues were considered homogeneous and isotropic, and the analysis was conducted exclusively based on a linear elastic behavior. Considering the model’s limitations, the FEA allowed obtaining results consistent with the clinical practice ones. The same model was used to simulate five situations of extraoral force application, and the comparative analysis of the results allows some important conclusions.

• Different lines of action of extraoral force combined with the Teuscher activator influence stress intensity and orthodontic and orthopedic force distribution in the maxilla.

• Stresses increased progressively when the force line of action moved in a posteroanterior direction.

• The extraoral force’s line of action used in Model 3 (30°) is the most compatible with the objectives of the hyperdivergent Class II malocclusion treatment in growing patients because it promotes displacement of the teeth and maxillary complex, promoting vertical control.

## References

[1] McNamara JA Jr (1981). Components of class II malocclusion in children 8-10 years of age. Angle Orthod.

[2] Buschang PH, Sankey W, English J (2002). Early treatment of hyperdivergent open-bite malocclusions. Semin Orthod.

[3] Teuscher U (1978). A growth-related concept for skeletal class II treatment. Am J Orthod.

[4] Wheeler TT, McGorray SP, Dolce C, Taylor MG, King GJ (2002). Effectiveness of early treatment of class II malocclusion. Am J Orthod Dentofacial Orthop. 2002;121:9-17. 
PMid:11786865Wheeler TT, McGorray SP, Dolce C, Taylor MG, King GJ. Effectiveness of early treatment of class II malocclusion. Am J Orthod Dentofacial Orthop.

[5] Singh GD, Thind BS (2003). Effects of the headgear-activator Teuscher appliance in the treatment of class II Division 1 malocclusion: a geometric morphometric study. Orthod Craniofac Res.

[6] Marsan G (2007). Effects of activator and high-pull headgear combination therapy: skeletal, dentoalveolar, and soft tissue profile changes. Eur J Orthod.

[7] Martins RP, da Rosa Martins JC, Martins LP, Buschang PH (2008). Skeletal and dental components of class II correction with the bionator and removable headgear splint appliances. Am J Orthod Dentofacial Orthop.

[8] Franchi L, Pavoni C, Faltin K Jr (2013). , McNamara JA Jr., Cozza P. Long-term skeletal and dental effects and treatment timing for functional appliances in class II malocclusion. Angle Orthod.

[9] Silvestrini BF, Lazzarotti L, Bini S, Migliorati M, Ugolini A (2020). Maxillary "en masse" high-pull traction in class II division 1 subjects: which kind of skeletal outcomes does it produce?. Eur J Paediatr Dent.

[10] Kang JM, Park JH, Bayome M, Oh M, Park CO, Kook YA (2016). A three-dimensional finite element analysis of molar distalization with a palatal plate, pendulum, and headgear according to molar eruption stage. Korean J Orthod.

[11] Jacob HB, Buschang PH, dos Santos-Pinto A (2013). class II malocclusion treatment using high-pull headgear with a splint: a systematic review. Dental Press J Orthod.

[12] Zervas ED, Galang-Boquiren MT, Obrez A, Costa Viana MG, Oppermann N, Sanchez F (2016). Change in the vertical dimension of class II division 1 patients after use of cervical or high-pull headgear. Am J Orthod Dentofacial Orthop.

[13] Nucera R, Militi A, Lo Giudice A, Longo V, Fastuca R, Caprioglio A (2018). Skeletal and dental effectiveness of treatment of class II malocclusion with headgear: a systematic review and meta-analysis. J Evid Based Dent Pract.

[14] Oh E, Ahn SJ, Sonnesen L (2020). Evaluation of growth changes induced by functional appliances in children with class II malocclusion: superimposition of lateral cephalograms on stable structures. Korean J Orthod.

[15] Gautam P, Valiathan A, Adhikari R (2009). Craniofacial displacement in response to varying headgear forces evaluated biomechanically with finite element analysis. Am J Orthod Dentofacial Orthop.

[16] Maruo IT, Maruo H, Saga AY, de Oliveira DD, Argenta MA, Tanaka OM (2016). Tridimensional finite element analysis of teeth movement induced by different headgear forces. Prog Orthod.

[17] Huiskes R, Chao EY (1983). A survey of finite element analysis in orthopedic biomechanics: the first decade. J Biomech.

[18] Knop L, Gandini LG Jr, Shintcovsk RL, Gandini MR (2015). Scientific use of the finite element method in orthodontics. Dental Press J Orthod.

[19] Tanne K, Hiraga J, Kakiuchi K, Yamagata Y, Sakuda M (1989). Biomechanical effect of anteriorly directed extraoral forces on the craniofacial complex: a study using the finite element method. Am J Orthod Dentofacial Orthop.

[20] Tanne K, Matsubara S, Sakuda M (1993). Stress distributions in the maxillary complex from orthopedic headgear forces. Angle Orthod.

[21] Chaves-Fernandes L, Farinazzo-Vitral RW, Yoshito-Noritomi P, Abrantes-Schmitberger C, da Silva Campos MJ (2019). Influence of the hyrax expander screw position on stress distribution in the maxilla: A study with finite elements. Am J Orthod Dentofacial Orthop.

[22] Freitas MR, Lima DV, Freitas KM, Janson G, Henriques JF (2008). Cephalometric evaluation of class II malocclusion treatment with cervical headgear and mandibular fixed appliances. Eur J Orthod.

[23] Mises Rv (1913). Mechanik der festen körper im plastisch-deformablen zustand. Nachrichten von der Gesellschaft der Wissenschaften zu Göttingen. Mathematisch-Physikalische Klasse.

[24] Türkkahraman H, Sayin MO (2006). Effects of activator and activator headgear treatment: comparison with untreated Class II subjects. Eur J Orthod.

[25] Bowden DE (1978). Theoretical considerations of headgear therapy: a literature review. 2. Clinical response and usage. Br J Orthod.

[26] Pfeiffer JP, Grobéty D (1982). A philosophy of combined orthopedic-orthodontic treatment. Am J Orthod.

[27] Teuscher U (1986). An appraisal of growth and reaction to extraoral anchorage. Simulation of orthodontic-orthopedic results. Am J Orthod.

[28] Jeong GM, Sung SJ, Chun YS, Mo SS (2009). Finite-element investigation of the center of resistance of the maxillary dentition. Korean J Orthod.

[29] Feizbakhsh M, Kadkhodaei M, Zandian D, Hosseinpour Z (2017). Stress distribution in maxillary first molar periodontium using straight pull headgear with vertical and horizontal tubes: a finite element analysis. J Dent Res.

[30] Alosman HS, Bayome M, Vahdettin L (2021). A 3D finite element analysis of maxillary molar distalization using unilateral zygoma gear and asymmetric headgear. Orthod Craniofac Res.

